# Bacterial phenotypic heterogeneity in DNA repair and mutagenesis

**DOI:** 10.1042/BST20190364

**Published:** 2020-03-20

**Authors:** Maxence S. Vincent, Stephan Uphoff

**Affiliations:** Department of Biochemistry, University of Oxford, South Parks Road, Oxford OX1 3QU, U.K.

**Keywords:** antibiotic resistance, DNA replication and recombination, DNA synthesis and repair, mutagenesis, phenotypic heterogeneity, single-cell analysis

## Abstract

Genetically identical cells frequently exhibit striking heterogeneity in various phenotypic traits such as their morphology, growth rate, or gene expression. Such non-genetic diversity can help clonal bacterial populations overcome transient environmental challenges without compromising genome stability, while genetic change is required for long-term heritable adaptation. At the heart of the balance between genome stability and plasticity are the DNA repair pathways that shield DNA from lesions and reverse errors arising from the imperfect DNA replication machinery. In principle, phenotypic heterogeneity in the expression and activity of DNA repair pathways can modulate mutation rates in single cells and thus be a source of heritable genetic diversity, effectively reversing the genotype-to-phenotype dogma. Long-standing evidence for mutation rate heterogeneity comes from genetics experiments on cell populations, which are now complemented by direct measurements on individual living cells. These measurements are increasingly performed using fluorescence microscopy with a temporal and spatial resolution that enables localising, tracking, and counting proteins with single-molecule sensitivity. In this review, we discuss which molecular processes lead to phenotypic heterogeneity in DNA repair and consider the potential consequences on genome stability and dynamics in bacteria. We further inspect these concepts in the context of DNA damage and mutation induced by antibiotics.

## Introduction

**Figure 1.**
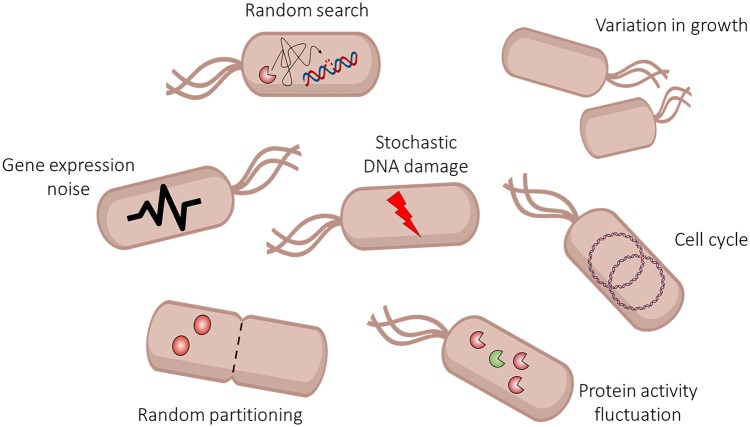
Sources of heterogeneity in DNA repair. Various molecular processes involved in DNA repair are inherently stochastic, including the random search of repair enzymes for lesions and fluctuations in the activity of individual enzymes. Gene expression noise and random partitioning of molecules at cell division create heterogeneity in the abundances of DNA repair proteins. Phenotypic variation in growth characteristics, cell morphology, or the cell cycle causes heterogeneity in the expression or functioning of the DNA repair system.

The maintenance of genome integrity is crucial for essential cell functions and accurate transfer of the genetic information across generations. For this reason, all organisms rely on protein machinery dedicated to protecting their DNA from alterations that can be caused by a variety of DNA damaging agents and processes that act on DNA [1]. Bacterial strains that lack DNA repair genes are often hypersensitive to DNA damage and exhibit elevated mutation rates. However, even a fully functional DNA repair system is not perfectly error-proof itself. The efficiency of DNA repair is limited by the stochastic nature of the underlying molecular events [2] (Figure 1). Lesions may be overlooked during the random search of a small number of DNA repair enzymes per cell or incorrectly processed due to interference by other cellular processes. Unrepaired DNA lesions hinder DNA replication, which can lead to replication stalling [3] and DNA double-strand breaks (DSB) [4], and cause DNA polymerases to incorporate mismatched nucleotides [5,6].

The DNA repair capacity per cell is governed by the concentrations of repair enzymes and available metabolic resources. Any fluctuations in these quantities can affect the ability of individual cells to repair DNA damage. Theoretical and experimental studies have demonstrated that gene expression is a stochastic process that is subject to noise [7,8]. Although gene regulatory feedback loops control the expression and activity of DNA repair proteins in ways that can buffer noise and equalise cells, each control layer also offers a potential mechanism of individualisation for single cells. In general, stochastic molecular events become manifest as phenotypic heterogeneity when the number of molecules or reactions per cell is small and each event has a large impact on cell function. Indeed, certain types of DNA lesions (such as DSBs) are rare [9–11] but highly toxic or mutagenic [4,12], and the copy numbers of important DNA repair proteins are very low in bacteria [13–15]. These considerations, supported by growing experimental evidence, suggest that phenotypic heterogeneity is a common feature of DNA repair systems. As a result, the same DNA damaging conditions may lead to different fates of individual bacteria in a population even when the cells are genetically identical and present in the same environment.

The mutation rate, like all traits, is subject to evolutionary selection governed by the opposing effects of beneficial and deleterious mutations on cell fitness [16]. On the one hand, most mutations have neutral or negative fitness effects in optimal growth conditions [17]. On the other hand, modification of DNA sequence is necessary for heritable adaptation to environmental challenges. In fact, bacterial strains isolated from natural environments and from infections frequently carry genetic alleles that increase mutation rates, e.g. via inactivation of DNA mismatch repair or replication proofreading [18,19]. Such mutator strains also arise during antibiotic treatment [20], and may accelerate the evolution of antibiotic resistance mutations. Following adaptation, mutator alleles tend to be maintained by genetic hitchhiking along with the associated adaptive mutations despite the increased rate of deleterious mutations [21]. Therefore, it could be beneficial for unicellular organisms to shift the balance between genome stability and plasticity temporarily according to their fitness in a given environment [18,22,23]. In principle, modulation of the mutation rate would allow cells to accelerate evolution when they are poorly adapted in their current environment without jeopardising the stability of a highly adapted genome during growth in optimal conditions [24]. At the centre of this duality, the activities of DNA repair and DNA replication fidelity mechanisms define the mutation rate in an individual cell. Heterogeneity in DNA repair generates subpopulations of cells that could transiently act as pools of increased genetic plasticity [15,24–26]. In this regard, phenotypic heterogeneity provides a source of genetic diversity, in an apparent reversal of the genotype-to-phenotype dogma.

Cell-to-cell variability and unsynchronised dynamics in DNA repair have long been undetectable due to the limitations of traditional genetic and biochemical assays that rely on bulk population measurements. However, a surge in developments of single-molecule and single-cell assays can now bypass these limitations, revealing origins and consequences of heterogeneity in DNA repair [27]. These tools include single-molecule tracking to follow protein motion and localisation *in vitro* and in living cells, super-resolution fluorescence microscopy to measure protein localisation relative to other cellular landmarks, and fluorescent reporters to quantify real-time gene expression dynamics. Notably, live-cell imaging has been revolutionised by the invention of various microfluidic devices that create defined growth environments suitable for monitoring and isolating single cells [28].

## Origins of DNA repair heterogeneity

The DNA molecule is sensitive to various forms of damage, arising spontaneously through loss or chemical modification of DNA bases and breaks in the DNA backbone [29], as well as exogenously from environmental DNA damaging agents. Genotoxins and mutagens that react with DNA include reactive oxygen and nitrogen species, alkylating agents and DNA cross-linking chemicals [1,30], and various types of protein toxins [31–33]. Environmental stress conditions such as starvation or antibiotic treatment can also alter a cell's metabolism in ways that lead to DNA damage [34–36]. To cope with these frequent insults, bacteria rely on DNA repair systems that are broad, interconnected and can be either versatile or specific to a type of damage. The amount and genomic location of DNA lesions will differ between cells in a population, leading to different repair pathway choices, heterogeneity in DNA damage responses, and ultimately diversity in cell fates.

### Stochastic events in DNA damage and repair

Depending on the type of lesion, DNA repair is performed in a single reaction (e.g. the direct repair reactions by photolyase or DNA methyltransferase enzymes) or in a pathway of reaction steps catalysed by a series of enzymes (e.g. in the case of base excision repair — BER, nucleotide excision repair — NER, mismatch repair — MMR, DSB repair by homologous recombination — HR). The first step in all these processes requires a repair factor to detect the damage site against a vast excess of undamaged DNA within the cell volume. Evidence from single-molecule imaging suggests that the lesion search of different types of repair enzymes involves facilitated diffusion through a combination of 3D Brownian motion and non-specific DNA binding with 1D sliding along DNA [37–39]. In addition to the intrinsic randomness of Brownian motion, it has been shown that some DNA repair enzymes switch stochastically between distinct DNA scanning modes with different diffusion coefficients during the search [40,41]. It is possible that random variation in the duration of the lesion search for single enzymes affects the probability of successful repair considering that the concentrations of DNA repair proteins are typically very low in bacteria, e.g. ∼1 Ada molecule per cell [15], ∼5 RecB and ∼5 RecC molecules [14], several hundred Pol1, Ligase [42] and UvrAB molecules [43]. The efficiency of lesion recognition is also influenced by the spatial distribution of the repair proteins within cells. Some repair proteins display a homogeneous random distribution within the nucleoid volume [42], but other repair factors are excluded from the nucleoid [43–45] and become recruited to DNA only in response to damage [43]. In the case of DSB repair HR, RecA proteins form a filament structure that can span the length of the bacterium and guides the homology search to match the broken DNA with an intact homologous sequence [45–47].

Following successful lesion recognition, enzymatic rates fluctuate over time and differ between individual enzymes [48]. Accordingly, *E. coli* RecBCD helicase-nuclease enzymes that initiate DSB repair display random variation in the DNA unwinding rate [49] due to equilibrium transitions between conformational sub-states [50]. These effects appear to be conserved as the *Mycobacterial* AdnAB helicase-nuclease also shows enzymatic heterogeneity with stochastic pauses at random sites on DNA [51]. Besides stochastic heterogeneity between chemically identical proteins, enzymatic heterogeneity can also be caused by ribosome mistranslations, which create a pool of mutated and truncated proteins with partial or complete loss of functionality [52]. An increase in the frequency of translation errors during cell stress can impair genome maintenance functions, leading to an increase in mutagenesis in a subpopulation of stressed cells [53].

Mutations arise due to the stochastic misincorporation of nucleotides by DNA polymerases [5]. The rates of DNA replication errors depend on a variety of factors, including the type of DNA polymerase, the template DNA sequence, and the efficiency of replication proofreading and DNA MMR mechanisms [54]. Changes in dNTP pools also affect replication fidelity by modulating the balance between DNA synthesis and the exonuclease proofreading rate of DNA polymerases [55]. Several studies showed that the replicative DNA polymerases and other replisome components exchange frequently at replication forks [56–60]. Therefore, the DNA polymerase composition of the replisome is likely more variable than previously anticipated, and potentially heterogeneous between cells. Because completion of DNA replication is necessary for cell survival and certain DNA base lesions block DNA synthesis by the replicative DNA polymerases, bacteria express alternative DNA translesion synthesis (TLS) polymerases that are capable of replicating past these lesions. However, TLS polymerases are considered mutagenic due to their lower base selection fidelity and a lack of proofreading activity compared with the replicative polymerases [6]. Although the concentrations of some TLS polymerases are comparable to the replicative polymerase in *E. coli*, these proteins do not localise at replication forks during normal growth [61,62] and contribute little to spontaneous mutagenesis [63]. But in the presence of DNA damage, TLS polymerases may replace replicative polymerases in the replisome by a controlled recruitment mechanism [64] or via stochastic protein exchange [65]. Alternative models suggest TLS polymerases act separately on DNA gaps left in the newly synthesised DNA behind the fork [3].

### Heterogeneous expression of DNA repair genes

Gene expression is inherently a stochastic process. Consequently, the number of proteins that are produced from a given gene fluctuates over time, which can lead to significant variation in protein expression levels between cells in a population [7,8]. Additional heterogeneity in protein abundances arises every time a cell divides and cytoplasmic molecules are partitioned randomly between the daughter cells [66]. Furthermore, DNA replication duplicates gene copies, causing cyclical changes in gene dosage [67]. These sources of gene expression heterogeneity are universal and therefore also affect DNA repair proteins including those of constitutively expressed housekeeping genes. To what extent the resulting heterogeneity in DNA repair capacity affects the actual rates of repair per cell depends on the level of DNA damage. In the case of BER, the reaction rates of DNA polymerase I and ligase enzymes showed little heterogeneity across cells despite significant variation in their expression levels [42]. This was likely the case because the activities of these enzymes were dictated by the rates of upstream reactions in the BER pathway [42].

In bacteria, it is common for DNA repair genes to be regulated by transcription factors and sigma factors via specific stress responses. Noise in stress sensing and signal transduction, together with feedback loops can amplify gene expression fluctuations and split cells into subpopulations with distinct stress response phenotypes. The broadly conserved SOS response comprises a large network of genes controlled by the LexA repressor, whose degradation is triggered via interaction with RecA [68]. The SOS regulon encodes crucial DNA repair proteins acting in DSB and NER pathways and error-prone DNA polymerases involved in TLS. Single-cell imaging of gene expression reporters showed that LexA-regulated genes display heterogeneous patterns of expression in normally growing cells and after DNA damage treatment [69–72]. Spontaneous SOS induction can result from the formation of DSBs that occur stochastically in a fraction of cells during unperturbed growth [9,10]. The adaptive response provides inducible protection against DNA alkylation damage [30,73]. Its main component, Ada, directly transfers damaging alkyl groups from DNA onto itself. Once alkylated, Ada becomes a transcriptional activator of its own gene, thereby triggering a positive feedback loop. Similar to many transcription factors [13], Ada is present at very low copy number in exponentially growing cells and a subpopulation of ∼30% of cells are devoid of a single Ada molecule [15,66]. These cells exhibit prolonged delays in the induction of the adaptive response after sudden exposure to alkylation damage [15]. Consequently, the population diversifies into two distinct subpopulations, marked by the induction of a strong adaptive response in cells that initially contained at least one Ada molecule and a failure to adapt for cells that have zero Ada molecules.

### Variation in other cellular processes influences DNA repair

In addition to noise that is intrinsic to the expression and function of the DNA repair system, heterogeneity in other cellular processes can propagate and influence DNA repair processes. The efficacy of DNA repair is linked to the DNA replication cycle and cell growth rate [74], which have been shown to fluctuate in single cells over time [75] and vary between cells in a population [76]. In most bacteria, DSB repair and the replication cycle are closely connected, as HR is restricted to regions of the chromosome that have been replicated. Although the presence of multiple chromosome copies is in principle beneficial for HR, hyper-initiation of replication can lead to the breakdown of replication forks [77,78]. Variability in the cell cycle also affects the expression of DNA repair genes, as in the case of the adaptive response where a reduced growth rate leads to a stronger response [79]. Furthermore, entry into stationary phase is associated with global changes in gene expression and metabolism, and modulates various DNA repair functions [80]. During active growth, cell age is also a potential factor contributing to heterogeneity in DNA damage and repair. It has been observed that the frequency of spontaneous SOS response induction increases in ageing but actively growing *E. coli* cells [81]. In turn, the DNA damage response can modulate the cell cycle, for example via inhibition of cell division by the SOS response.

Overall, the aforementioned examples underscore that cell-to-cell heterogeneity in DNA damage levels and DNA repair activities can arise from the stochastic nature of the damage itself, the rates at which individual repair proteins localise lesions and fix them, and the connections between DNA repair and other cellular processes. Gene expression noise is a general source of cellular heterogeneity that affects DNA damage recognition, damage response regulation, and repair capacity in single cells.

## Consequences of DNA repair heterogeneity

### Variation in cell mortality

Heterogeneity in the expression of DNA repair genes has been linked to the survival probabilities of individual cells in response to DNA damage. For example, delayed activation of the adaptive response is associated with decreased survival in a subpopulation of cells that have a transient lack of DNA repair capacity after exposure to DNA alkylation damage [15,82]. Cell-to-cell variation in the expression of DNA repair proteins by the SOS response correlates with survival of antibiotic stress [83] and is linked to the recovery of persister cells after antibiotic treatment [84,85]. Insufficient DNA repair capacity is clearly detrimental for cells, but an overabundance or misregulation of many repair proteins also causes toxicity [15,86]. In principle, gene expression noise can perturb a fine-tuned genome maintenance system. For instance, an imbalance of proteins that act together in a DNA repair pathway causes accumulation of incompletely processed DNA damage sites. This has been demonstrated for the BER pathway where overexpression of DNA glycosylases breaks the coordination of reaction steps, leading to the formation of toxic and mutagenic repair intermediates in cells [87]. Maladaptive BER is also responsible for the toxicity of the antibiotic trimethoprim due to the combined effects of thymidine depletion and oxidative stress [88]. The lack of thymidine results in the formation of single-stranded DNA gaps during replication, and the excision of oxidised DNA bases from single-stranded DNA generates lethal DSBs.

### Variation in mutagenesis

**Figure 2.**
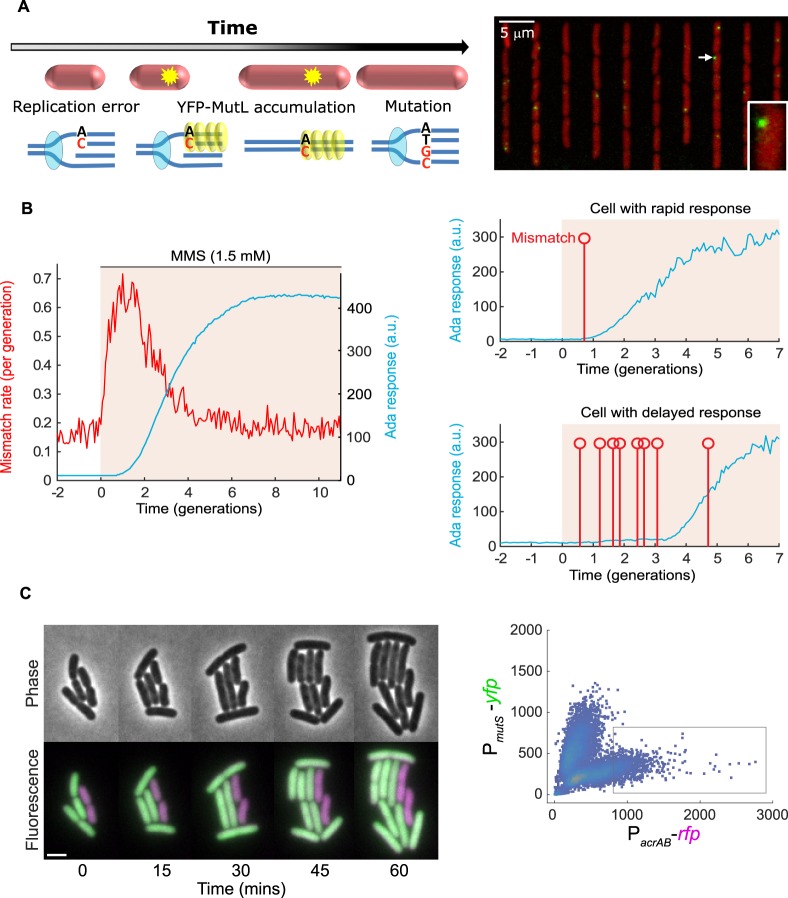
Visualising the effects of DNA repair heterogeneity on mutagenesis. (**A**) Nascent mutations can be detected in single cells by microscopy. Schematic (left image) and snapshot of *E. coli* cells in microfluidic channels (right image). Foci of the MMR protein MutL labelled with a YFP fluorescent protein mark nascent DNA replication errors (Reproduced from [17] with permission from AAAS). (**B**) Monitoring real-time dynamics of mutagenesis in response to DNA alkylation damage treatment (MMS). Left panel: Slow induction of the Ada response (blue line) creates a transient lack of DNA repair capacity in *E. coli*. This causes a pulse in the frequency of DNA mismatches (red line, measured using a similar approach as in panel A). Right panels: Heterogeneity in the timing of the Ada response dictates the duration of the mutation pulse in single cells. Cells with a delayed Ada response experience more DNA mismatches (red vertical markers) than cells with a rapid response (Adapted from [82] with permission from PNAS). (**C**) Linking mutagenesis and cellular heterogeneity in antibiotic tolerance. Time-lapse microscopy reveals that expression of the antibiotic efflux pump AcrAB (purple) is associated with a decreased expression of the MMR gene *mutS* (green) in single *E. coli* cells (Reproduced from [26] with permission from AAAS).

Cell-to-cell variation in DNA repair and DNA replication processes can cause mutation rate heterogeneity within a clonal cell population. This is exemplified by the phenomenon of stress-induced mutagenesis, where starvation conditions lead to increased mutation rates due to overexpression of error-prone TLS polymerases (in *E. coli* particularly Pol IV) [89]. This hypermutation phenotype appears to be limited to a subpopulation of cells that exhibit elevated SOS response expression following the formation of a spontaneous DSB [12]. Although DSB repair by HR is in principle error-free, utilisation of TLS polymerases makes the process mutagenic. Besides the SOS response, other gene regulatory systems including the RpoS general stress response and the stringent response contribute to this effect [89]. Describing the impact of phenotypic heterogeneity on mutation rates in bacteria has long been a very complex task due to a number of technological challenges. Traditional bulk genetics assays (i.e. based on different types of fluctuation tests) rely on selectable phenotypes, such as antibiotic or phage resistant mutants, to estimate mutation rates [75,77]. The assays can be biased in neglecting cell death events [90] or polyploidy effects [91] and do not report on genome-wide mutation rates. Mutation accumulation experiments combined with whole-genome sequencing overcome some of these limitations [63], but neither technique is able to directly resolve cell-to-cell heterogeneity and dynamics in mutagenesis. Single-cell imaging now provides the possibility to visualise the formation of fluorescently labelled DNA mismatches as a proxy to monitor mutagenesis in real-time [17,82,92] (Figure 2A). In *E. coli*, ∼1% of replication errors are overlooked by the MMR system [93] and converted into stable mutations at a basal rate of approximately one in a thousand cell generations [63,93]. Imaging DNA mismatches confirmed that mutations occur randomly at a constant rate during unperturbed growth, such that the number of mutations per cell and the waiting times between mutation events follow ‘memoryless' Poisson statistics [17,92].

Exposure to DNA damaging agents or loss of DNA repair and replication fidelity genes is associated with an increase in mutagenesis. The DNA repair system can become ineffective or saturated in such conditions [80,94–96]. Cell cycle timing, population density, and nucleoid compaction also appear to modulate mutation rates in bacteria [16,93,97,98]. Imaging of DNA mismatches enables correlating mutagenesis with any other phenotypic characteristic (e.g. the abundance of particular DNA repair proteins) in real-time at the single-cell level. This approach showed that stochastic activation of the adaptive response to DNA alkylation damage results in a pulse of mutagenesis [82]. The pulse predominantly affects a subpopulation of cells that experience delayed adaptation to the stress (Figure 2B). Considering that infection of macrophages is associated with DNA alkylation stress [99], such an effect may also contribute to the dynamics of mutagenesis inside a host.

## Antibiotic-induced mutagenesis

Combatting the rise of antibiotic resistance in bacterial pathogens has become one of the most urgent priorities in biomedical research [100]. Accumulating evidence from multiple laboratories highlight a major conundrum in the management of bacterial infections: antibiotics which are effective at killing pathogens also promote mutagenesis and thereby accelerate the evolution of drug resistance [101–103]. In many cases, antibiotic-induced mutagenesis has been linked to the SOS response [85,104] and expression of TLS polymerases [102,103,105,106]. In support of this, single-cell imaging showed a strong temporal correlation between the induction of the SOS response and an increased rate of DNA mismatches during fluoroquinolone treatment [82]. Furthermore, fluoroquinolone treatments have also been shown to stimulate horizontal gene transfer [107,108]. Inhibition of central metabolic processes by diverse types of bactericidal antibiotics (including aminoglycosides, fluoroquinolones, trimethoprim, and beta-lactams), even at low concentration, leads to the formation of reactive oxygen species [34,35,101,109], which cause mutagenic base lesions and DNA breaks.

Drug-sensitive bacteria are able to withstand antibiotics at low doses owing to tolerance mechanisms that are controlled by a variety of stress responses. Interestingly, some genetic regulators of antibiotic stress responses also control the expression of DNA repair genes. The MarA transcription factor modulates expression of porins, efflux pump, and lipid trafficking genes but also activates Exonuclease VII under fluoroquinolone treatment [110]. Direct cross-talk between antibiotic tolerance and mutagenesis has been demonstrated for the antibiotic efflux pump AcrAB, whose heterogeneous expression is inversely correlated with the expression of MMR genes [26] (Figure 2C). Cells that transiently induce AcrAB are less sensitive to antibiotics and display elevated mutagenesis at the same time [26]. The existence of this phenomenon may be explained by the notion that antibiotic-induced mutagenesis increases the chance of resistance but at the same time jeopardises the fitness of most cells [16]. Therefore, cell populations may exploit phenotypic heterogeneity in DNA repair to restrict mutagenesis to a subpopulation of cells while maintaining the genome of most cells. Indeed, it has been shown that fluoroquinolone treatment triggers a differentiation of bacteria via heterogeneous induction of the SOS response and the RpoS general stress response [24]. These cells grow into filaments containing multiple chromosome copies that facilitate genetic diversity via mutagenic repair of ciprofloxacin-induced DSBs. Antibiotic resistance mutations can also originate in persister cells that induce error-prone TLS polymerases when they resume DNA replication after the antibiotic stress is over [85].

Based on the aforementioned and other reports, DNA repair and stress response factors are considered promising targets for novel therapeutics aimed at inhibiting the evolution of resistance mutations [101,103,111]. These targets include the regulators of the SOS response RecA and LexA [111], the RpoS general stress response [24], and the Mfd translocase of the transcription-coupled NER pathway [112–114].

## Future directions

The rise of single-molecule techniques and quantitative live-cell imaging allows interrogating DNA repair processes with unprecedented resolution that reveals molecular stochastic effects and cell-to-cell heterogeneity [27]. Such technologies, combined with advances in microfluidics and image analysis [28,115], and a growing arsenal of functional markers and reporters available to visualise specific DNA lesions, repair intermediates, and DNA damage responses open opportunities to examine the role of noise in many DNA repair processes that have so far been studied only at a bulk population level. Evidently, the origin of some phenotypic variation is truly stochastic, i.e. caused by unpredictable molecular fluctuations in cells, while other variable cell behaviours may appear random but are actually the result of an underlying deterministic process that was invisible in an experiment. The distinction is not always clear-cut, and future simultaneous imaging of multiple intracellular and environmental reporters will help to pinpoint sources of phenotypic heterogeneity in DNA repair and mutagenesis. A range of unsolved questions relates to the consequences of DNA repair heterogeneity for cell function. Is the heterogeneity a by-product of inaccurate regulation or does it provide a fitness advantage that was selected during evolution? The presence of heterogeneity in a range of different stress responses in diverse bacterial species suggests the latter answer may be true, but direct proof for this remains hard to produce. Ample evidence shows that antibiotics increase mutation rates, but it has been challenging to pinpoint the exact function of stress responses in this context. For instance, although theoretical work indicates evolutionary benefits of mutation rate variability [116] it has been suggested that the main benefit of the SOS response lies in its effect on cell survival rather than mutagenesis [117]. Notably, stress-induced mutagenesis is not limited to bacteria, but also seen in cancer cells and unicellular eukaryotes [22,118,119], reflecting the high degree of functional conservation of DNA repair and replication mechanisms throughout evolution. Overall, the question whether heterogeneity in DNA repair and mutagenesis is beneficial for populations from an evolutionary point of view remains open and will need to be addressed through a combination of experimental and theoretical approaches.

## Perspectives

- **Importance of the field:** Noise in the DNA repair system can perturb genome maintenance but can also be a source of cell-to-cell heterogeneity that generates genetic diversity in bacterial populations.
- **Current thinking:** Gene expression noise and other stochastic processes cause heterogeneity in the abundances and activities of DNA repair proteins amongst isogenic cells. Variation in DNA repair affects the mortality and rates of mutagenesis of individual cells.
- **Future directions:** Novel single-cell assays open possibilities for interrogating the sources and consequences of heterogeneity in DNA repair and mutagenesis, including its role in antibiotic tolerance and resistance evolution.

## Open Access

Open access for this article was enabled by the participation of the University of Oxford in an all-inclusive Read & Publish pilot with Portland Press and the Biochemical Society under a transformative agreement with JISC.

## Abbreviations

BER: base excision repair
DSB: double-strand breaks
HR: homologous recombination
MMR: mismatch repair
NER: nucleotide excision repair
TLS: translesion synthesis
